# Immune Checkpoint Inhibitors and Endocrine Disruption: A Case of Hyponatremia and Adrenal Insufficiency

**DOI:** 10.7759/cureus.70089

**Published:** 2024-09-24

**Authors:** Shashwat Kafley, Silbin Tamrakar, Mohammed Samra, Suriya Shanmugar, Isha Gupta

**Affiliations:** 1 General Practice, Enam Medical College and Hospital, Dhaka, BGD; 2 Internal Medicine, University of Debrecen, Debrecen, HUN; 3 Internal Medicine, ACS Medical College and Hospital, Chennai, IND; 4 Nephrology, Middletown Medical, Middletown, USA; 5 Nephrology, Garnet Health Medical Center, Middletown, USA; 6 Internal Medicine/Nephrology, Touro College of Osteopathic Medicine, Middletown, USA

**Keywords:** adrenal insufficiency, electrolyte imbalance, hypokalemia, hyponatremia, immune checkpoint inhibitors, malignant melanoma, oncology, pembrolizumab

## Abstract

Immune checkpoint inhibitors, such as pembrolizumab, have transformed cancer therapy by enhancing the immune system's ability to combat tumors, but they can also lead to immune-related adverse events, including adrenal insufficiency.

This case report presents a 52-year-old male with a history of malignant melanoma who developed adrenal insufficiency after four months of pembrolizumab therapy. The patient was admitted with symptoms of malaise, vomiting, abdominal pain, and poor appetite. Laboratory tests revealed significant metabolic abnormalities including hyponatremia, hypokalemia, and elevated thyroid-stimulating hormone. Further endocrine evaluation confirmed the diagnosis of secondary adrenal insufficiency, likely due to pembrolizumab-induced inflammation of the pituitary gland. The patient was treated with corticosteroid replacement therapy, leading to clinical improvement, and pembrolizumab was discontinued due to the risk of worsening adrenal insufficiency.

This case underscores the importance of early recognition and management of adrenal insufficiency in patients receiving pembrolizumab. While the exact cause of pembrolizumab-induced adrenal insufficiency is not fully understood, it may involve an autoimmune attack on cells in the pituitary gland. Prompt identification and treatment are essential to prevent potentially life-threatening complications, and this case highlights the need for clinicians to maintain a high level of awareness for adrenal insufficiency in patients presenting with nonspecific symptoms during or after immunotherapy.

## Introduction

Pembrolizumab is a humanized monoclonal immunoglobulin (Ig)G4 antibody that targets the programmed death-1 (PD-1) receptor, a key immune checkpoint protein on activated T cells. By binding to PD-1 and preventing its interaction with its ligands, PD-L1 and PD-L2, pembrolizumab inhibits the negative regulation of T-cell activation, thereby enhancing the immune system's ability to mount an anti-tumor response. This mechanism of action has led to its widespread use in the treatment of various malignancies, including non-small cell lung cancer, colorectal cancer, bladder cancer, endometrial cancer, and prostate cancer [1].

Despite its efficacy, pembrolizumab is associated with immune-related adverse events (irAEs), which can impact patient outcomes. Among these irAEs, hypothyroidism is the most common, but rarer and potentially life-threatening conditions, such as hypophysitis, can also occur [2]. Hypophysitis, observed in less than 1% of patients receiving anti-PD-1 antibodies, can lead to secondary adrenal insufficiency (AI) and hypopituitarism [3]. This condition often results from an autoimmune attack on corticotrophs, the cells responsible for producing adrenocorticotropic hormone (ACTH), leading to ACTH deficiency [4].

Secondary AI, arising from ACTH deficiency due to hypophysitis, presents with non-specific symptoms, making it challenging to diagnose without endocrine function testing. While the initial management of AI involves corticosteroid replacement, the optimal approach for monitoring AI after starting pembrolizumab therapy remains a subject of ongoing debate [5]. Here, we present a case of a 52-year-old male who developed AI while being treated with pembrolizumab for melanoma.

## Case presentation

A 52-year-old male was admitted to the hospital with a three-day history of non-projectile vomiting, generalized dull abdominal pain, and a several-week history of poor appetite and malaise. His medical history was significant for hypertension, hyponatremia, migraine, and malignant melanoma status post-excision. He had been receiving pembrolizumab therapy for the past four months as part of his melanoma treatment.

Upon admission, laboratory tests revealed severe hyponatremia with a sodium level of 121 mEq/L (reference range: 136-144 mEq/L) and hypokalemia with a potassium level of 3.3 mEq/L (reference range: 3.5-5.1 mEq/L). Further evaluation indicated an elevated thyroid-stimulating hormone level of 39.9 mU/L (reference range: 0.5-5 mU/L). In light of these findings, consultations were obtained from endocrinology for possible adrenal insufficiency and elevated thyroid stimulating hormone levels, gastroenterology for his vomiting, nausea, and abdominal pain, nephrology for his electrolyte abnormalities and oncology for his significant past history of melanoma during his stay in the hospital. Following this, a multi-specialty coordinated effort was carried out for his treatment. Serum cortisol levels and ACTH stimulation tests were done according to the endocrinologist's order. Salt tablets and fluid restriction to 1200 ml fluid per day were done as per nephrology consultation. The patient’s daily electrolyte levels were also monitored, as shown in Table 1. Laboratory results at the time of admission and discharge are presented in Table 1.

**Table 1:** Laboratory values for blood and urine tests at admission and discharge ACTH: Adrenocorticotropic hormone; CBC: Complete blood count.

	Laboratory values at admission	Laboratory values at discharge	Reference values	Units
CBC with differentials				
White Blood Cell	5.4	5.1	4.3 - 10.6 * 10^3	/microLitre
Red Blood Cell	5.69	-	4.30 - 5.80 * 10^6	/microLitre
Hemoglobin	14.6	14.1	13.3 - 17.0	g/dL
Hematocrit	43.6	42.6	40.3 - 50.3	%
Mean Corpuscular Volume	76.6	82.6	81.4 - 99.0	femtolitre (fL)
Mean Coposcular Hemoglobin	25.7	26.2	26.8 - 32.9	picogram (pg)
Mean Corpuscular Hemoglobin Concentration	33.5	31.7	31.4 - 34.9	g/dL
Red Cell Distribution Width	20.3	22.5	11.6 - 14.9	%
Platelets	250	313	132 - 337 * 10^3	/microLitre
Complete Metabolic Panel				
Sodium	121	138	136 - 144	mEq/L
Potassium	3.3	3.2	3.5 - 5.1	mEq/L
Chloride	85	103	101 - 111	mEq/L
Carbon Dioxide	24	26	22 - 32	mEq/L
Blood Urea Nitrogen	8	11	8 - 20	mg/dL
Creatinine	1.16	1.18	0.55 - 1.02	mg/dL
Glucose	99	84	70 - 99	mg/dL
Protein	6.6	7.1	6.4 - 8.3	g/dL
Albumin	3.9	4.1	3.5 - 4.8	g/dL
Calcium	9.3	9.5	8.5 - 10.1	mg/dL
Alkaline Phosphatase	47	44	45 - 117	U/L
Alanine transaminase	106	91	15 - 41	U/L
Aspartate transaminase	174	112	17 - 63	U/L
Creatine Kinase total	3765	599	49 - 397	U/L
Urine analysis				
Urine Sodium Random	49	-	20	mEq/L
Urine Potassium Random	27.3	-	20	mEq/L
Thyroid Stimulating Hormone	39.911	-	0.5-5	mU/L
Thyroglobulin antibody	73	-	< or = 1	IU/mL
Thyroid Peroxidase antibody	53	-	< 9	IU/mL
ACTH stimulation test	< 5	-	6 - 50	pg/mL
Cortisol, total serum	1.4 (morning) 1.7 (evening)	-	4 - 22 (morning) 3 - 17 (evening)	mcg/dL

Subsequent doses of pembrolizumab were held due to the patient’s elevated liver enzymes and the known association of pembrolizumab with adrenal insufficiency. A computed tomography scan of the abdomen and pelvis was performed, which ruled out the possibility of adrenal hemorrhage.

Morning and evening serum cortisol levels were measured the following day, with the morning value reported at 1.4 mcg/dL (reference range: 4-22 mcg/dL) and the evening value at 1.7 mcg/dL (reference range: 3-17 mcg/dL). An ACTH stimulation test was also performed, revealing decreased ACTH production, further supporting the diagnosis of secondary adrenal insufficiency. The patient’s symptoms were attributed to his ongoing immunotherapy. Consequently, the patient was started on salt tablets, intravenous fluids, levothyroxine, 0.5 mg of dexamethasone, and 50 mcg of fludrocortisone.

The patient was continuously monitored in the hospital, where he made a gradual recovery. His sodium and potassium levels stabilized at 138 mEq/L and 3.2 mEq/L, respectively. He was discharged on the prescribed medications. Daily sodium and potassium levels are presented in Table 2.

**Table 2:** Daily serum sodium and potassium values

Date	Sodium (136 - 144 mEq/L)	Potassium (3.5 - 5.1 mEq/L)
07/07/2024	121	3.3
07/08/2024	124	3.2
07/09/2024	127	3.9
07/10/2024	129	4.0
07/12/2024	137	3.6
07/16/2024	138	3.2

## Discussion

In this case, we explore the well-documented side effects of pembrolizumab, an immune checkpoint inhibitor, specifically focusing on its association with adrenal insufficiency leading to hyponatremia. Pembrolizumab enhances antitumor responses by binding to the PD-1 receptor, thereby preventing cancer cells' PD-L1 receptors from suppressing the immune system [6]. 

This case report highlights the development of adrenal insufficiency in a patient treated with pembrolizumab, contributing to the evolving understanding of irAEs associated with immune checkpoint inhibitors. Pembrolizumab-induced hyponatremia may result from adrenal insufficiency and hypothyroidism. Hypothyroidism can cause decreased cardiac output, leading to antidiuretic hormone release and subsequent water retention [7]. AI can be due to pituitary hypophysitis leading to secondary adrenal insufficiency, or it can directly affect the adrenal glands. Its pathogenesis is largely unknown, but it may be linked to autoimmunity targeting corticotrophs [8,9]. AI can lead to dehydration, hyponatremia, hyperkalemia, changes in blood count (such as anemia, eosinophilia, and lymphocytosis), and hypoglycemia [10].

Management of AI requires hormone replacement therapy, including both glucocorticoids and mineralocorticoids, with the dose tailored to severity of the condition. In severe cases, intravenous or intramuscular administration is needed. Immunotherapy must be stopped until the patient achieves clinical stability [11].

Although there is no established protocol for monitoring cortisol levels, this case highlights the importance of early screening for AI when patients present with symptoms suggestive of AI. While the median onset of AI is around 10 weeks, it can occur months after the cessation of treatment. Prompt treatment initiation is critical to prevent adrenal crisis and reduce the risk of mortality [6,9].

## Conclusions

In summary, adrenal insufficiency (AI) caused by pembrolizumab, which can lead to hyponatremia, is an uncommon but potentially severe immune-related adverse event that necessitates prompt identification and treatment. This condition can manifest during active treatment or even months after pembrolizumab therapy has ended, underscoring the importance of ongoing vigilance in patients with a history of using immune checkpoint inhibitors.

The clinical signs are often non-specific and may include fatigue, nausea, dizziness, and electrolyte disturbances. Diagnosis requires a high degree of clinical suspicion and should be confirmed by evaluating cortisol levels and performing ACTH stimulation tests.

Management of pembrolizumab-induced AI involves the immediate initiation of corticosteroid replacement therapy, which typically results in rapid symptom relief. Long-term care may include hormone replacement therapy and educating patients about stress dosing and emergency protocols.

As the use of immune checkpoint inhibitors grows in cancer treatment, healthcare providers need to be mindful of potential endocrine side effects like AI. Early detection, appropriate treatment, and continuous follow-up are key to achieving the best possible outcomes for patients undergoing pembrolizumab or similar therapies.
